# Efficacy and tolerability of darunavir/r 600/100 mg bid in treatment-experienced patients: 48-week data from a German outpatient cohort

**DOI:** 10.1186/1758-2652-13-S4-P28

**Published:** 2010-11-08

**Authors:** J van Lunzen, D Gorriahn, T Wünsche, S Dupke, P Gute, CK Schewe, B Ranneberg

**Affiliations:** 1 Universitätsklinikum Eppendorf, Ambulanzzentrum des UKE, Bereich Infektiologie, Hamburg, Germany; 2 Private Practice, München, Germany; 3 Private Practice, Berlin, Germany; 4 Praxis Driesener Strasse, Berlin, Germany; 5 Infektiologikum Frankfurt, Frankfurt, Germany; 6 ICH Hamburg, Hamburg, Germany; 7 Janssen, Johnson&Johnson-Platz 1, Neuss, Germany

## Background

Darunavir/Ritonavir (DRV/r) dosed 600/100 mg bid has shown good efficacy and tolerability in treatment-experienced patients in clinical trials (POWER, TITAN). To assess whether these results can be transferred to clinical practice and a more diverse patient population this prospective non-interventional Janssen-sponsored cohort study was established.

## Methods

Between August 2007 and March 2009, 340 HIV-1 infected antiretroviral-experienced patients from 62 German centers enrolled in this non-interventional cohort. All patients received DRV/r 600/100 mg bid as part of their HAART. Virological and immunological response was monitored every 3 months. Patients were followed for up to 48 weeks.

## Results

296/340 (87%) patients were male with a mean age of 47 years (SD 9.9); median time since HIV diagnosis was 12 years. At baseline, mean viral load (VL) was 4.7 log_10_ copies/mL (SD 5.44) with a mean CD4 count of 392 cells/mm^3^ (SD 251); 213 (63%) patients started therapy with CD4 < 200 cells/mm^3^. Patients had been pretreated with NRTIs (94%), PIs (88%), NNRTIs (63%), FI (11%), INI (5%) and/or CCR5 (3%). No data is available for 17 patients. At baseline, only 138 patients received DRV/r in combination with 2 NRTIs; the majority (202/340) of patients received DRV/r in different combinations based on the individual pretreatment situation. Of note, 116 patients received a combination containing DRV/r, RAL + other antiretrovirals. ETR and DRV/r plus other ARVs were used in 23 patients. 51 patients received an NRTI-sparing regimen. At the time of this analysis, 32 patients had discontinued prematurely and 251/340 patients had reached week 48. VL and CD4 measurements at week 48 are available for 186 patients, 80.6 % patients achieved a VL < 50 copies/ml (VL < 400 copies/ml: 94.6 %), mean CD4 count in these patients (n=185) increased from 402 at baseline to 476 cells/ mm^3^ (SD: 262; p <0.001). 10 patients (2.9%) discontinued DRV/r due to adverse events. Virological failure (VL >50 copies/mL at 2 visits) was documented in 10 patients (2.9%). 3 patients were genotyped; primary PI mutations were detected in all 3 patients, DRV-RAMs in none of them.

## Conclusions

Overall, Darunavir/r, dosed at 600/100 mg bid in combination with other antiretrovirals, showed good efficacy and safety in treatment-experienced patients in clinical practice.

